# Validation of the Vanderbilt Holistic Face Processing Test

**DOI:** 10.3389/fpsyg.2016.01837

**Published:** 2016-11-23

**Authors:** Chao-Chih Wang, David A. Ross, Isabel Gauthier, Jennifer J. Richler

**Affiliations:** ^1^Office of Institutional Research, National Central UniversityZhongli, Taiwan; ^2^Department of Psychology, National Chung Cheng UniversityChia-Yi, Taiwan; ^3^Department of Psychological and Brain Sciences, University of Massachusetts Amherst, AmherstMA, USA; ^4^Department of Psychology, Vanderbilt University, NashvilleTN, USA

**Keywords:** VHPT, holistic processing, individual differences, face recognition, measurement

## Abstract

The Vanderbilt Holistic Face Processing Test (VHPT-F) is a new measure of holistic face processing with better psychometric properties relative to prior measures developed for group studies (Richler et al., 2014). In fields where psychologists study individual differences, validation studies are commonplace and the concurrent validity of a new measure is established by comparing it to an older measure with established validity. We follow this approach and test whether the VHPT-F measures the same construct as the composite task, which is group-based measure at the center of the large literature on holistic face processing. In Experiment 1, we found a significant correlation between holistic processing measured in the VHPT-F and the composite task. Although this correlation was small, it was comparable to the correlation between holistic processing measured in the composite task with the same faces, but different target parts (top or bottom), which represents a reasonable upper limit for correlations between the composite task and another measure of holistic processing. These results confirm the validity of the VHPT-F by demonstrating shared variance with another measure of holistic processing based on the same operational definition. These results were replicated in Experiment 2, but only when the demographic profile of our sample matched that of Experiment 1.

## Introduction

People extract a wealth of socially relevant information from a single face, such as identity, emotional expression (reviewed in Bruce and Young, 1986; Calder and Young, 2005), gender (e.g., O’Toole et al., 1998), and even personality traits, such as trustworthiness (e.g., Oosterhof and Todorov, 2008). Holistic processing—the tendency to process faces as wholes rather than collections of features—is involved in each of these types of judgments (identity: reviewed in Richler and Gauthier, 2014; emotional expression: Calder et al., 2000; Tanaka et al., 2012; gender: Zhao and Hayward, 2010; trustworthiness: Todorov et al., 2010), suggesting that holistic processing plays a critical role in face perception.

A variety of tasks have been used to measure holistic processing (cf. Richler et al., 2012), but the composite task (Young et al., 1987; Hole, 1994; Farah et al., 1998) is arguably the most common (Richler and Gauthier, 2014). In the composite task, participants are asked to judge whether one half (e.g., top) of two sequentially presented composite faces are the same or different while ignoring the other task-irrelevant half (e.g., bottom). Holistic processing is inferred from a congruency effect: performance is better on congruent trials (both target and irrelevant parts are the same or both are different) than incongruent trials (one part same, the other part different)–participants cannot ignore the task-irrelevant part because faces are processed as wholes.

After decades of research on face recognition, researchers have begun to turn to the study of individual differences in this area (reviewed in Yovel et al., 2014). However, because face recognition research has a history rooted in group studies, the measures that are common in the literature for assessing different constructs relevant to face recognition and perception may not be suited for measurement of individual differences. To be useful for individual differences research, a measure has to demonstrate good internal consistency (correlation across different test items or trials), and if the concept is thought to be stable, it should also demonstrate test–retest reliability. The composite task is highly sensitive in group studies (13 subjects needed for 95% power, *p* = 0.05, Richler and Gauthier, 2014), but this same measure has very low internal consistency (∼0.2; DeGutis et al., 2013; Ross et al., 2015). Indeed, the power of standard statistical tests is not necessarily related to the reliability of a dependent variable (Nicewander and Price, 1983). Critically, if a measure has little systematic variance, it is unreasonable to expect it to share variance with other measures, and any observed correlations are difficult to interpret. Although “disattenuated” correlations (what the correlation would be if reliability was perfect, Nunnally, 1970), can be computed, disattenuated estimates can overcorrect and be imprecise (i.e., have large confidence intervals, Wetcher-Hendricks, 2006).

We developed the Vanderbilt Holistic Face Processing Test (VHPT-F; Richler et al., 2014), the first test of holistic face processing designed specifically for use in individual differences research. The VHPT-F is modeled after the composite task: subjects are instructed to selectively attend to part of a face and holistic processing is indexed by an inability to do so. However, unlike the composite task where a single test face is presented that requires a same-different response, in the VHPT-F three faces are presented at test, and participants must select the face that contains the target part. In addition to using a three-alternative forced choice design that reduces guessing and increases scale range, our main strategy to improve reliability was to target a broader range of holistic ability across trials. In the composite task, like most cognitive measures, all trials target roughly the same level of difficulty, usually chosen to avoid floor and ceiling performance. The result is an effect that only discriminates among subjects whose ability is within a narrow (typically average) range. Therefore, in the VHPT-F we added variability in the extent to which different trials would best discriminate various levels of the holistic ability continuum by varying the size of the target part relative to the whole face. Trials where the target part is a very small part of the face (e.g., eyes only) should be processed holistically by most subjects except those with the least holistic tendencies (e.g., those who are best able to selectively attend to face parts), while trials where the target part is a very large part of the face (e.g., top 2/3) should not be processed holistically except by those with the most holistic tendencies (e.g., those who are the least able to selectively attend to face parts). Group-level data confirmed that this manipulation had the intended effect: holistic effects were largest when face parts were small and selective attention was more difficult, and smallest when face parts were large and selective attention was easier (Richler et al., 2014).

The VHPT-F has higher internal consistency (0.56) than the composite task, and measures a stable trait (test–retest reliability of 0.49, *r*_disattenuated_ = 0.94, after a 6 month delay). The VHPT-F produces very large average effect size for holistic processing ($\eta_{p}^{2}$ = 0.75) and is normally distributed in a normal adult population (Richler et al., 2014). While this previous work demonstrates that the VHPT-F has good psychometric properties, it is important to verify that it measures the expected construct, beyond the most basic face validity. In fields where psychologists study individual differences, measurement validation studies are commonplace, but this is not yet standard practice in cognitive and perceptual areas. The concurrent validity of a scale like the VHPT-F can be established by comparing it to the more standard composite task, which has been used in an extensive literature on holistic face processing (reviewed in Richler and Gauthier, 2014).

While these two tasks are similar in many ways, including the same operationalization of holistic processing as a failure of selective attention, they may not necessarily correlate. For example, the VHPT-F measures selective attention for a much broader range of parts than the standard composite task. Some argue that top and bottom trials should not be combined in the composite task because the effect is larger for top than bottom judgments (Rossion, 2013), but the correlation of holistic processing across different parts has not been reported before. Finally, the VHPT-F and composite task differ in response format (three-alternative forced choice on identity versus same-different judgment), which may change the strategy participants adopt.

In Experiment 1, we tested the validity of the VHPT-F by having participants complete the VHPT-F and composite task. There were two composite task blocks, one where the top face half was the target, and one where the bottom face half was the target. The correlation between top and bottom composite task trials sets a reasonable upper limit for the correlation, we could expect across different tasks, as this is a correlation for the same task and the same faces; only the attended half differs. Note that even if the magnitude of holistic processing differs for different attended parts (cf. Rossion, 2013), this would not influence whether or not holistic processing correlates across them. Participants also completed the Vanderbilt Expertise Test for cars (VET-car; McGugin et al., 2012) to test if holistic processing of faces is related to expertise with another category. For instance, it may be that those who are the most holistic apply a similar strategy to other object categories, resulting in better performance. This analysis was exploratory, and is only minimally reported here.

## Experiment 1

### Method

#### Participants

All participants were recruited in accordance with approval of Vanderbilt University Institutional Review Board. Out of a pool of 1000 participants who performed the VET-car on Amazon Mechanical Turk (see Lee et al., 2015), we sent 384 invitations to male participants^1^stating they were eligible for new tasks and would receive a bonus if they completed them all. These tasks included the composite task with top judgments (completed by 195 participants), the composite task with bottom judgments (completed by 181 participants), the VET-bird (completed by 180 participants, but these data were not analyzed because several trials were repeated by mistake), and the VHPT-F (completed by 174 participants).

In total 166 subjects completed all tasks. We discarded data from 30 participants: 20 participants failed to follow instructions on the VHPT-F and did not view the study face for the entire 2 s on more than 25 trials; 8 participants failed 2/3 easy VHPT-F practice trials; 1 participant was an extreme univariate outlier on the VHPT-F (7 SD above the mean); finally, we screened for multivariate outliers in the correlation between holistic processing for top and bottom judgments in the composite task, and 1 participant had an externally studentized residual greater than 3. Therefore, data from 136 participants (mean age = 34 years, 107 Caucasian, 14 Asian, 7 African American, 6 Hispanic/Latino, 1 Pacific Islander, 1 race not disclosed) are included in the analyses.

#### VET-Car

The Vanderbilt Expertise Test was created as a battery of domain-specific tests to measure object recognition ability in different domains (McGugin et al., 2012). We used the car subtest, as used in VanGulick et al. (2016). Subjects studied images of six cars without labels for as long as they chose. Subjects then selected the car that corresponded to one of the six studied targets in 48 3-alternative forced choice trials (target image and two foils). In the first 12 trials, the studied image of one target appeared with two foils, and feedback was provided (correct/incorrect). The last 36 trials used targets that were different examples of the same studied cars, presented without feedback.

#### Vanderbilt Holistic Face Processing Test (Version 2.0)

**FIGURE 1:**
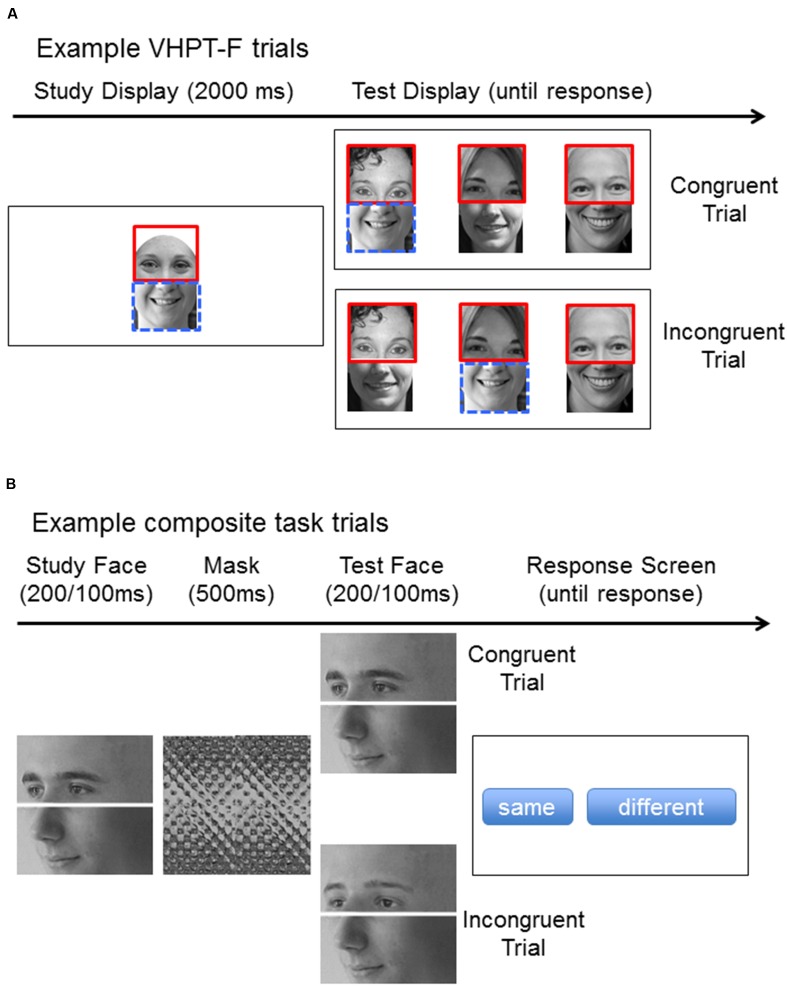
**(A)** Example VHPT-F trials where the top face half is the target. The correct response is the face on the left. On congruent trials, the target part is paired with the distractor as during study. On incongruent trials, the target part is paired with a new distractor part, and the distractor part from the study face is paired with a foil (the task-irrelevant part of the study face is outlined in blue here for illustrative purposes only). **(B)** Example composite task trials. In these examples, the bottom is the target part and the correct response is “same.” On congruent trials, the target and distractor face halves are associated with the same response (“same” in this example). On incongruent trials, the face halves are associated with different responses (in this example, the bottom half is “same” but the top half is “different”).

On each trial (see **Figure 1A**), a study composite face was shown for 2 s followed by a test display with three composite faces. Participants were instructed to select the composite face containing the target part with the same identity (but different image) as the target part in the study composite, while ignoring the rest of the face. The target part was outlined in red at study and test. The correct target part was paired with either the same distractor parts (congruent trials) or different distractor parts (incongruent trial) relative to study (see **Figure 1A**). There were nine blocks of 20 trials, each with a different target part (top 2/3, bottom 2/3, top half, bottom half, top 1/3, bottom 1/3, eyes, mouth, nose), for a total of 180 trials^2^. On 116 trials, face composites were created from grayscale male and female faces obtained from the internet. The faces used to create composites on a given trial were either all male or all female. On the remaining 64 trials, face composites were created from grayscale computer-generated faces. Both real and computer-generated trials were used to increase variability in trial types, which benefits reliability. Holistic processing scores are calculated using accuracy for congruent trials minus that for incongruent trials.

#### Composite Task

Because previous work found that the composite task was most reliable when a small set of stimuli (five tops and five bottoms) were used to create composites (Ross et al., 2015), composite task stimuli were created from the top half of five Caucasian male faces and the bottom halves of five other Caucasian males from the CVL Face Database^3^ (Peer, 1999; Solina et al., 2003) presented from a three-quarter view (135^°^ rotation^4^). The five face tops and bottoms were randomly combined to create composites (169 × 175 pixels). A white line four pixels thick separated face halves so it was unambiguous where the top half ended and the bottom half began.

Each composite task (top and bottom judgments) included 80 trials (see **Figure 1B**). The study and test composite faces were shown for 200 ms in the first 40 trials, and 100 ms in the second 40 trials. Presentation time was reduced in the second half of trials to add variability in trial difficulty, which is beneficial for test reliability. Otherwise, the trials were identical, with a mask for 500 ms (and a test image for 200 ms). Participants indicated if the target half (top or bottom) of the test composite was the same or different by clicking on the appropriate response option displayed on the screen. Each block of 40 trials included 10 trials for each combination of response (same/different) and congruency (congruent/incongruent).

### Results

**FIGURE 2:**
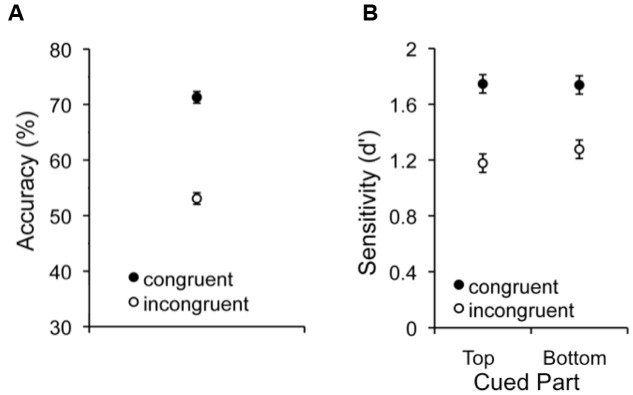
**Mean performance for congruent and incongruent trials in the** **(A)** VHPT-F and **(B)** composite task. Error bars show 95% confidence intervals for within-subject effects (Loftus and Masson, 1994).

Mean performance in the VHPT-F and composite task are shown in **Figure 2**. A repeated measures ANOVA on VHPT-F accuracy with congruency (congruent/incongruent) as a factor revealed significant holistic processing (better performance on congruent vs. incongruent trials; *F*_1,135_ = 423.36, *MSE* = 33.82, *p* < 0.001, $\eta_{p}^{2}$ = 0.76). A 2 (congruency: congruent/incongruent) × 2 (target part: top/bottom) repeated measures ANOVA on d’ in the composite task also revealed significant holistic processing (better performance on congruent vs. incongruent trials; *F*_1,135_ = 151.81, *MSE* = 0.24, *p* < 0.001, $\eta_{p}^{2}$ = 0.53), and the magnitude of holistic processing did not differ between top ($\eta_{p}^{2}$ = 0.44) and bottom ($\eta_{p}^{2}$ = 0.38) target parts (*F*_1,135_ = 2.67, *MSE* = 0.15, *p* = 0.10, $\eta_{p}^{2}$ = 0.02).

Reliability for all tasks is shown in **Table 1**. The correlation between the congruency effects (d’ for congruent – d’ for incongruent) across top and bottom composite task trials was *r*_134_ = 0.24. This value approaches the reliability of each congruency effect individually (see **Table 1**), which suggests a considerable amount of shared variance when the correlation is disattenuated (*r*_disattenuated_ = 0.73, *p* < 0.01). The correlation between holistic processing in the composite task and VHPT-F was *r*_134_ = 0.27 (*r*_disattenuated_ = 0.57, *p* < 0.01). These correlations were similar when only Caucasian subjects (*n* = 107) were included (top and bottom composite task trials: *r*_105_ = 0.27; composite task and VHPT-F: *r*_105_ = 0.26). Holistic processing in the VHPT-F was equally correlated with holistic processing in top and bottom composite task trials (top: *r*_134_ = 0.21, *r*_disattenuated_ = 0.49, *p* < 0.05; bottom: *r*_134_ = 0.21, *r*_disattenuated_ = 0.60, *p* < 0.05)^5^. There was no evidence for any relationship between car expertise measured by the VET-car and holistic processing in any task (all *rs* < 0.06, *r*_disattenuated_ < 0.1).

**Table 1:** Reliability for the composite task and VHPT-F (Guttman’s λ2) and the VET-car (Cronbach’s α) in Experiment 1.

Task	All trials	Top trials	Bottom trials
Composite task	0.48	0.40	0.27
VHPT-F	0.47		
VET-car	0.88		


### Discussion

The composite task that is frequently used to measure holistic processing in group studies often has low internal consistency (∼0.2; DeGutis et al., 2013; Ross et al., 2015), which limits its utility for the study of individual differences. However, given the central role of holistic processing measured in the composite task in the literature (reviewed in Richler and Gauthier, 2014), it is important to confirm that a new holistic processing measure (VHPT-F; Richler et al., 2014) taps into the same construct.

Reliability is not a property of tasks but of measurements (Thompson, 1994), and the reliability of the VHPT-F measurements in the present sample is somewhat lower than what was obtained in prior work (Richler et al., 2014), although it is still higher than the typical reliability of measurements in the standard composite task (Ross et al., 2015). Serendipitously, the reliability of our VHPT-F scores was matched in the present work by the reliability of those in the standard composite task. This is likely because, we implemented a version of the composite task with a small number of face halves, based on prior evidence that using fewer faces in the composite task increases reliability (Ross et al., 2015). Note, however, that for the purpose of correlating holistic processing with other tasks, stimulus repetition may be problematic (see Richler et al., 2015). Stimulus repetition has been shown to influence experimental measures in several domains (e.g., Malley and Strayer, 1995; Endress and Potter, 2014), and it can introduce spurious contributions in the measure, such as the ability to learn from repeated presentations and sensitivity to proactive interference (Underwood, 1957). Most importantly, stimulus repetition could introduce spurious correlations between the composite task where stimuli repeat and other tasks that also include stimulus repetition. Here, this did not seem to be the case across domains with the VET-car, which repeats cars, and it could not have inflated the correlation with the VHPT-F, because this task does not repeat stimuli. However, it could have inflated the correlation between top and bottom half judgments. Indeed, contributions from stimulus repetition may explain some of the non-shared variance between the composite task, where stimuli were repeated, and the VHPT-F, where stimuli did not repeat.

Despite the differences in format, holistic processing measured in the VHPT-F was significantly correlated with holistic processing measured in the composite task, with the disattenuated correlation suggesting approximately 40% shared variance. Observing a correlation between the composite task and VHPT-F may seem obvious in retrospect, because there are many similarities between the tasks (e.g., instructions to selectively attend to parts of composite faces). However, the tasks differ in many important ways, such that if, we had not found a correlation, we could have pointed to several factors as explanation. For example, differences in task format (e.g., three-alternative forced choice vs. same-different judgment) could have led to very different task strategies. Moreover congruency effects do not consistently correlate across various versions of the Stroop task (Salthouse and Meinz, 1995; Ward et al., 2001; Shilling et al., 2002; Yehene and Meiran, 2007) – if they did, we would not be surprised because they are different versions of the same task, but had this not been tested, we would not know that in fact they often do not.

Importantly, the correlation between holistic processing in the VHPT-F and composite task was similar to the correlation between holistic processing on top and bottom composite task trials, that is, a correlation between holistic processing measured with the same faces, in the same task^6^. This suggests that holistic processing measured in the VHPT-F and composite task is correlated to the extent that holistic processing correlates within the composite task itself, across top and bottom judgments. While some have proposed that holistic processing should only be measured with top part judgments to maximize the size of the effect (Rossion, 2013), our results reveal a relatively strong but not perfect relationship between holistic processing for different parts. The shared variance is large, but the correlation is far from perfect even for the same exact faces, which underlines the importance of measuring holistic processing with several face parts (as in the VHPT-F). This is because to the extent that one wishes to adequately capture individual differences in the application of “holistic face processing” on a single measure, the content validity of the test will depend on sampling the entire domain over which it applies (Cronbach, 1971; Aiken, 1979).

## Experiment 2

Because, we wanted to include an exploratory analysis of the relationship between performance with cars and holistic face processing, and prior work showed that car and face processing were differentially related in men and women (McGugin et al., 2012), we only recruited male participants in Experiment 1; this limits our conclusions about task validity. In addition, in Experiment 1 faces were presented in a three-quarter view in the composite task, but in frontal views in the VHPT-F, which may have limited correlations between them. Thus, the first goal of Experiment 2 was to replicate Experiment 1 with both men and women and with a frontal-view composite task.

In addition, the VHPT-F is based on the congruency measure of holistic processing. However, a large literature has used an alternative measure of holistic processing in the composite task, the alignment effect (see Rossion, 2013 for a review). According to this measure, holistic processing is reflected by higher accuracy on same-incongruent misaligned versus aligned trials: the different task-irrelevant half makes it more difficult to identify the target half as “same,” but this effect is reduced when the face configuration is disrupted by misalignment. This measure of holistic processing has been criticized for tracking response biases unrelated to holistic processing (see Richler and Gauthier, 2013, 2014 for reviews). Consistent with this view, the meta-analytic effect sizes for the alignment effect and congruency effect measures of holistic processing were not significantly correlated across 27 studies (*r*_25_ = 0.27, *p* = 0.18), suggesting that they are not in fact measuring the same thing. However, a more optimal comparison would be to test the correlation between measures of holistic processing across participants. Thus, in Experiment 2, we included misaligned trials in the composite task so, we could assess the correlation between two different holistic processing measures in the composite task that have been widely used in group studies.

### Methods

#### Participants

Two hundred participants who completed the VHPT-F on Amazon Mechanical Turk in exchange for $1.75 were invited to complete the composite task for an additional $2. A total of 121 participants completed both tasks. Data from one participant were discarded for failing to follow instructions on the VHPT-F, and data from an additional five subjects were discarded for below chance performance in at least one condition in the composite task. Therefore, data from 115 participants (mean age = 37.6 years, 86 Caucasian, 6 Asian, 15 African American, 5 Hispanic/Latino, 3 race not disclosed) are included in the analyses.

#### Vanderbilt Holistic Face Processing Test (Version 2.1)

The VHPT-F 2.1 was used in Experiment 2. This version is identical to version 2.0 used in Experiment 1, except trials that were programmed incorrectly were fixed. In addition, based on item analyses of data from 525 subjects, 16 trials that were not correlated with overall condition scores (e.g., congruent trials that were negatively correlated with overall performance on congruent trials) were replaced or modified.

#### Composite Task

The composite task was identical to Experiment 1, with the following exceptions. Stimuli were the top halves of five female faces and the bottom halves of five different male faces from the Max Planck Institute database that were converted to grayscale.

On misaligned trials, the study face was aligned, and the top and bottom face halves of the test face were offset such that the edge of one half fell in the center of the other half. The target part was always the top face half. Because the alignment effect measure of holistic processing is calculated based on data from only a quarter of the total trials, we included more trials in Experiment 2 to increase the reliability of this measure. There were 40 trials for each combination congruent/incongruent, aligned/misaligned, and same/different for a total of 320 trials.

### Results

**FIGURE 3:**
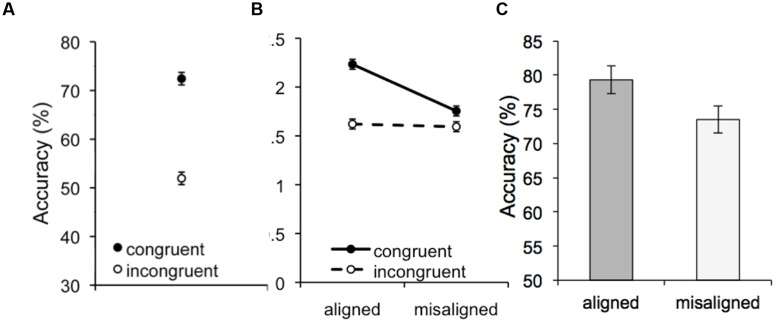
**Mean performance for all holistic processing measures.** **(A)** Accuracy on congruent and incongruent trials in the VHPT-F. **(B)** Sensitivity (d’) as a function of congruency and alignment in the composite task. **(C)** Accuracy for same-incongruent aligned and misaligned trials in the composite task. Error bars show 95% confidence intervals for within-subject effects (Loftus and Masson, 1994).

Mean performance for all holistic processing measures is shown in **Figure 3**. A repeated measures ANOVA on VHPT-F accuracy with congruency (congruent/incongruent) as a factor revealed significant holistic processing (better performance on congruent vs. incongruent trials; *F*_1,114_ = 495.59, *MSE* = 48.72, *p* < 0.001, $\eta_{p}^{2}$ = 0.81). A 2 (congruency: congruent/incongruent) × 2 (alignment: aligned/misaligned) repeated measures ANOVA on d’ in the composite task also revealed significant holistic processing: performance was better on congruent versus incongruent trials (*F*_1,114_ = 110.19, *MSE* = 0.16, *p* < 0.001, $\eta_{p}^{2}$ = 0.49) and this congruency effect was significantly larger on aligned versus misaligned trials (*F*_1,114_ = 75.57, *MSE* = 0.08, *p* < 0.001, $\eta_{p}^{2}$ = 0.40). There was also a significant main effect of alignment (*F*_1,114_ = 76.16, *MSE* = 0.10, *p* < 0.001, $\eta_{p}^{2}$ = 0.40).

Finally, although a repeated-measures ANOVA on accuracy on same-incongruent trials revealed a significant effect of alignment (*F*_1,114_ = 16.25, *MSE* = 119.64, *p* < 0.001, $\eta_{p}^{2}$ = 0.13), this effect is in the opposite direction than theoretically predicted, with better performance on aligned than misaligned trials. In other words, the alignment effect measure did not show evidence of holistic processing at the group level.

Reliability for all measures is shown in **Table 2**. The alignment effect and congruency effect^7^ measures of holistic processing were significantly correlated (*r*_113_ = 0.46, *r*_disattenuated_ = 0.71, *p* < 0.001). Surprisingly, however, neither composite task measure of holistic processing was significantly correlated with the VHPT-F (congruency effect: *r*_113_ = 0.16, *r*_disattenuated_ = 0.26, *p* = 0.10; alignment effect: *r*_113_ = 0.15, *r*_disattenuated_ = 0.27, *p* < 0.10).

**Table 2:** Reliability for both composite task measures of holistic processing and the VHPT-F (Guttman’s λ2) in Experiment 2.

Task	Measure	
Composite task		
	Congruency effect	0.59
	Alignment effect	0.71
VHPT-F		0.60


Given that one of the goals of Experiment 2 was to recruit a more diverse sample than Experiment 1, we tested whether differences in demographic variables between samples could explain the failure to replicate correlations between the composite task and VHPT-F measures of holistic processing.

In Experiment 1, we only tested male participants. When, we restricted our analyses in Experiment 2 to only male participants (*n* = 47), we found significant correlations between holistic processing in the VHPT-F and both composite task measures that were comparable in magnitude to the correlations in Experiment 1 (congruency effect: *r*_45_ = 0.29, *r*_disattenuated_ = 0.49^8^, *p* < 0.05; alignment effect: *r*_45_ = 0.35, *r*_disattenuated_ = 0.49, *p* < 0.05). These correlations were not significant for female participants (*n* = 68; congruency effect: *r*_66_ = 0.05, *r*_disattenuated_ = 0.08, *p* = 0.68; alignment effect: *r*_66_ = 0.05, *r*_disattenuated_ = 0.08, *p* = 0.68). However, differences between correlations in male and female participants were not significant (*zs* < 1.6, *ps* > 0.10). The congruency effect and alignment effect in the composite task were significantly correlated in both male (*r*_45_ = 0.54, *r*_disattenuated_ = 0.83, *p* < 0.001) and female (*r*_65_ = 0.43, *r*_disattenuated_ = 0.66, *p* < 0.001) participants.

**FIGURE 4:**
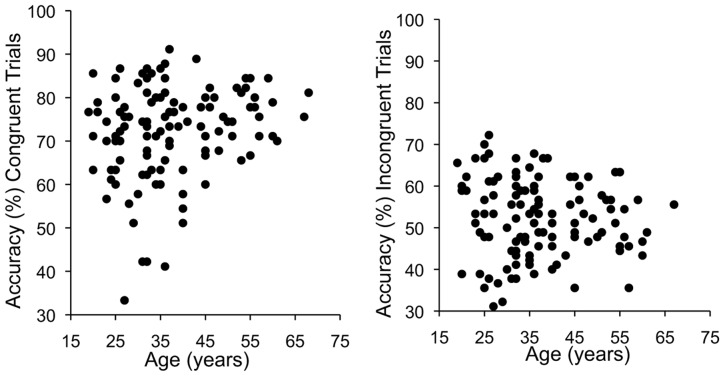
**Correlation between age (years) and accuracy on congruent **(Left)** and incongruent **(Right)** VHPT-F trials**.

We also found significant differences in the age distribution between Experiment 1 and Experiment 2 (*t*_239_ = 2.80, *p* < 0.01), with more older participants in Experiment 2. As can be seen in **Table 3**, age was only significantly correlated with the VHPT-F holistic processing measure. Notably, this effect was not present in Experiment 1 (*r*_134_ = 0.10, *p* = 0.27), and is larger than in a previous study (*r*_219_ = 0.19, Richler et al., 2014). Examination of scatterplots for correlations between age and VHPT-F performance (**Figure 4**) suggest a severe restriction in range for congruent trials in participants older than 45. When, we restricted our analyses to participants 45 years old or younger (*n* = 87), we found a significant correlation between the congruency effect measure of holistic processing in the composite task and the VHPT-F that is comparable in magnitude to that observed in Experiment 1 (*r*_85_ = 0.26, *r*_disattenuated_ = 0.44, *p* = 0.02). In this restricted sample, the correlation between the congruency effect and alignment effect measures was *r*_85_ = 0.48 (*r*_disattenuated_ = 0.74, *p* < 0.001), and the correlation between the alignment effect measure and the VHPT-F was not significant (*r*_85_ = 0.14, *r*_disattenuated_ = 0.22, *p* = 0.2). Mean-level effects for all subsamples (male, female, 45 years, or younger) were qualitatively the same and quantitatively very similar as for the full sample (see **Table 4**).

**Table 3:** Correlations between holistic processing measures and age.

Task	Age
VHPT-F	0.34^∗^
Congruency effect	-0.04
Alignment effect	-0.13


*Significant correlations (*p* < 0.05) indicated by asterisks.*

**Table 4:** Mean performance and effect size ($\eta_{p}^{2}$) for each measure of holistic processing for each subsample analyzed in Experiment 2.

Measure	Sample	Mean 1	Mean 2	$\eta_{p}^{2}$	*p*
VHPT-F		Congruent	Incongruent		

	Male	71.80	50.61	0.81	<0.001
	Female	72.88	52.86	0.81	<0.001
	≤45 years	71.06	52.12	0.79	<0.001

Congruency effect		Congruent	Incongruent		

	Male	2.17	1.60	0.49	<0.001
	Female	2.28	1.63	0.63	<0.001
	≤45 years	2.25	1.59	0.62	<0.001

Alignment effect		Misaligned	Aligned		

	Male	73.16	79.47	0.19	0.002^∗^
	Female	73.76	79.23	0.10	0.010^∗^
	≤45 years	72.95	78.30	0.10	0.002^∗^


*Means show accuracy (%) for the VHPT-F and Alignment Effect, and d’ for the Congruency Effect. Note that for each measure holistic processing is indexed by higher performance in the Mean 1 vs. Mean 2 condition. ^∗^significant but in the opposite direction than theoretically predicted.*

### Discussion

The correlation between holistic processing in the composite task and the VHPT-F found in Experiment 1 did not replicate in Experiment 2, although the correlations were small in both experiments (Experiment 1 = 0.27, Experiment 2 = 0.16) and the difference between them is not significant (*z* = -0.9, *p* = 0.37). The samples differed in age and sex distributions, and when we restricted our sample to be more similar to Experiment 1, the results replicated. This suggests that there may be subject properties that mediate performance in these measures, particularly the VHPT-F. In a prior report, the VHPT-F showed a particularly large holistic processing effect in women older than 45 [relative to younger women and to men regardless of age (Richler et al., 2014)]. Neither that sex effect nor the present one were expected, nor are they similar. While it is difficult to explain the sex effect, the age effect could be due to a selection confound, whereby the older people who participate on AMT may be a particularly motivated or high-performing subset. The correlation between the VHPT-F and age was not found Experiment 1, and was numerically smaller in a previous study (*r*_221_ = 0.19, Richler et al., 2014). However, the proportion of participants older than 45 years was larger in Experiment 2 (24%) than in these previous studies (16% in Experiment 1, 20% in Richler et al., 2014). Prior work does show a number of interactions between face characteristics and the demographics of the population tested (Malpass and Kravitz, 1969; Wright and Stroud, 2002; Wiese et al., 2013; Zebrowitz et al., 2015). More work is needed to determine whether this age effect in the VHPT-F is driven by a true change in cognitive ability, a selection bias in older adults, or whether there is no more than a spurious restriction of range in the present sample.

On average, we observed a reverse alignment effect (better performance in aligned than misaligned trials). This replicates findings in a study (*n* = 101) that also measured the alignment effect (hit rate for same-incongruent trials) in the context of the composite design used to measure congruency effects (complete design) with different face stimuli and a larger set of face parts (Ross et al., 2015). A meta-analysis of the alignment effect in 28 studies that used the complete composite task found the effect to be small ($\eta_{p}^{2}$ = 0.14, 95% CI: 0.07, 0.22) and the effect to be significant in only 25% of the studies (Richler and Gauthier, 2014). Whether this is true only when the alignment effect is measured in the context of the complete design, suggesting it is highly sensitive to context, or whether the report of alignment effects has been overestimated due to publication bias, we do not know.

With regards to individual differences in the alignment effect, its correlation with the congruency effect was consistent regardless of sample, although it may be somewhat inflated compared to the correlations with the VHPT-F because the two measures were derived from the same dataset. Indeed, this is suggested by the fact that the alignment effect did not correlate with the VHPT-F in either the full sample, or when we restricted the sample based on age. These results are consistent with prior suggestions that the alignment effect lacks validity (Cheung et al., 2008; Richler et al., 2011a,b,c).

## General Discussion

Here, we validated the VHPT-F by demonstrating that it correlates with another measure of holistic processing based on the same operational definition, the composite task. Admittedly, this correlation was quite small because the standard composite task measure comes with a lot of measurement noise and is generally ill-suited for individual differences. When a measure has low reliability, it limits how it can correlate with other measures, even if they are more reliable. Ideally, we would have followed the approach taken in fields like intelligence, where new tests are compared and validated against other established tests. Unfortunately, the reality is that there is not another measure of holistic processing that has been designed for the purpose of measuring individual differences, so there is no test with established validity and reliability to validate against. While, we could have used another standard measure from group studies, such as the part-whole task, there is little evidence that different operational definitions of holistic processing should tap into the same construct, as they may reflect different co-occurring but independent mechanisms (Richler et al., 2012). Another approach would be to use a measure of face recognition or inversion effects to validate the VHPT-F. But, recent evidence suggests that while holistic processing is routinely observed for faces but not objects in novices (e.g., Farah et al., 1998; Richler et al., 2011c), it does not relate to face recognition performance (Richler et al., 2015), which is instead predicted by performance with parts (Royer et al., 2015; Sunday et al., in submission), and whether upright and inverted faces rely on qualitatively different mechanisms is debated (e.g., Rossion and Boremanse, 2008 vs. Richler et al., 2011c). Indeed, trying to find a way to validate the VHPT-F reveals how little, we truly know about holistic processing, but it is exactly these kinds of questions that motivated us to pursue creation of reliable measures in the first place.

Our results lead to the following conclusions. The alignment effect in the composite task lacks validity as a measure of holistic processing. The congruency effect in the composite task is more stable in its average effects, and when faces are repeated (as in the versions of the composite task used here) it produces measurements that are more reliable than with large sets of face parts (Richler et al., 2015; Ross et al., 2015). For the purpose of validating the VHPT-F, it was justifiable to use a few face version of the composite task because (1) we absolutely needed some reliability, and (2) the VHPT-F does not have stimulus repetition, so this could not inflate observed correlations between them (see Richler et al., 2015), although admittedly face repetition may have added noise to the composite task measure that ultimately reduced correlations with other measures. But, for future work, we advocate the use of the VHPT-F that achieves similar levels of reliability (0.5–0.6) without repeating faces. This is important to ensure that in future work correlations with holistic processing are not due to face learning or other factors related to stimulus repetition (see Richler et al., 2015). We provided evidence that the VHPT-F measures the same sort of holistic processing that is measured in the standard composite task, despite a number of procedural differences. Future studies with much larger samples with the VHPT-F will be needed to establish useful age and sex norms.

## Author Contributions

C-CW and JR contributed to the design and study concept. All authors contributed to collect the data, the data analysis, and interpretation. JR drafted the early manuscript, and C-CW provided the revised version. DR and IG provided critical comments. IG replied to the reviewers.

## Conflict of Interest Statement

The authors declare that the research was conducted in the absence of any commercial or financial relationships that could be construed as a potential conflict of interest.

## Footnotes

1 Only male participants were recruited because prior work showed that car and face processing were differentially related in men and women (McGugin et al., 2012).

2 Due to a programming error, data from 1 congruent to 5 incongruent trials were excluded from the analyses.

3 The face images used in this part have been provided by the Computer Vision Laboratory, University of Ljubljana, Slovenia (http://www.lrv.fri.uni-lj.si/facedb.html).

4 This view was chosen because the stimuli were created for a separate project in which car composites were also used. In pilot work for that project, these faces produced a clear composite effect as typically obtained with front-view images. Prior work with a different version of the composite task also found the same effect for front, profile, and three-quarter views (McKone, 2008).

5 Note that even though raw correlations are identical, disattenuated correlations differ because of differences in reliability for top and bottom trials.

6 The raw correlation for holistic processing on top and bottom composite task trials was numerically smaller than the correlation between holistic processing in the composite task and VHPT-F. However, the disattenuated correlation was larger due to the lower reliability of bottom trials.

7 When misaligned trials are included in the composite task, holistic processing can be indexed by regressing out the congruency effect misaligned trials from the congruency effect on aligned trials (e.g., DeGutis et al., 2013). However, previous work has shown very little shared variance between congruency effects on aligned and misaligned trials, such that using the regression measure generally produces highly similar results to the congruency effect on aligned trials only (Richler et al., 2014, 2015). Indeed, here the regression measure and congruency effect on aligned trials only are almost perfectly correlated (*r*_115_ = 0.94, *p* < 0.001). Therefore, for simplicity and consistency with Experiment 1, we used the congruency effect on aligned trials only as the congruency effect composite task measure.

8 Disattenuated correlations in the analyses with restricted samples use the reliability estimate from the largest possible sample, we have (**Table 2**).

## References

[B1] Aiken L. R. (1979). *Psychological Testing and Assessment.* Boston, MA: Allyn & Bacon.

[B2] Bruce V. Young A. (1986). Understanding face recognition. *Br. J. Psychol.* 77 305–327. 10.1111/j.2044-8295.1986.tb02199.x 3756376

[B3] Calder A. J. Young A. W. (2005). Understanding the recognition of facial identity and facial expression. *Nat. Rev. Neurosci.* 6 641–651. 10.1038/nrn1724 16062171

[B4] Calder A. J. Young A. W. Keane J. Dean M. (2000). Configural information in facial expression perception. *J. Exp. Psychol. Hum. Percept. Perform.* 26 527–551. 10811161 10.1037//0096-1523.26.2.527

[B5] Cheung O. S. Richler J. J. Palmeri T. J. Gauthier I. (2008). Revisiting the role of spatial frequencies in the holistic processing of faces. *J. Exp. Psychol. Hum. Percept. Perform.* 34 1327–1336. 19045978 10.1037/a0011752

[B6] Cronbach L. J. (1971). “Test validation,” in *Educational Measurement*, 2nd Edn, ed. Thomdike R. L. (Washington, DC: American Council on Education).

[B7] DeGutis J. Wilmer J. Mercado R. J. Cohan S. (2013). Using regression to measure holistic face processing reveals a strong link with face recognition ability. *Cognition* 126 87–100. 10.1016/j.cognition.2012.09.004 23084178

[B8] Endress A. D. Potter M. C. (2014). Large capacity temporary visual memory. *J. Exp. Psychol. Gen.* 143 548–565. 10.1037/a0033934 23937181 PMC3974584

[B9] Farah M. J. Wilson K. D. Drain M. Tanaka J. N. (1998). What is “special” about face perception? *Psychol. Rev.* 105 482–498. 10.1037/0033-295X.105.3.482 9697428

[B10] Hole G. J. (1994). Configurational factors in the perception of unfamiliar faces. *Perception* 23 65–74. 10.1068/p230065 7936977

[B11] Lee W.-Y. Cho S.-J. McGugin R. W. Van Gulick A. B. Gauthier I. (2015). Differential item functioning analysis of the vanderbilt expertise test for cars (VETcar). *J. Vis.* 15:23 10.1167/15.13.23 PMC4588597 26418499

[B12] Loftus G. R. Masson M. E. J. (1994). Using confidence intervals in within-subject designs. *Psychol. Bull. Rev.* 1 476–490. 10.3758/BF03210951 24203555

[B13] Malley G. B. Strayer D. L. (1995). Effect of stimulus repetition on positive and negative identity priming. *Percept. Psychophys.* 57 657–667. 10.3758/BF03213271 7644326

[B14] Malpass R. S. Kravitz J. (1969). Recognition for faces of own and other race. *J. Pers. Soc. Psychol.* 13 330–334. 10.1037/h0028434 5359231

[B15] McGugin R. W. Richler J. J. Herzmann G. Speegle M. Gauthier I. (2012). The vanderbilt expertise test reveals domain-general and domain-specific sex effects in object recognition. *Vis. Res.* 69 10–22. 10.1016/j.visres.2012.07.014 22877929 PMC3513270

[B16] McKone E. (2008). Configural processing and face viewpoint. *J. Exp. Psychol. Hum. Percept. Perform.* 34 310–327. 10.1037/0096-1523.34.2.310 18377173

[B17] Nicewander W. A. Price J. M. (1983). Reliability of measurement and the power of statistical tests: some new results. *Psychol. Bull.* 94 524–533. 10.1037/0033-2909.94.3.524

[B18] Nunnally J. C. (1970). *Introduction to Psychological Measurement.* New York, NY: McGraw-Hill.

[B19] Oosterhof N. N. Todorov A. (2008). The functional basis of face evaluation. *Proc. Natl. Acad. Sci. U.S.A.* 105 11087–11092. 10.1073/pnas.0805664105 18685089 PMC2516255

[B20] O’Toole A. J. Deffenbacher K. A. Valentin D. McKee K. Huff D. Abdi H. (1998). The perception of face gender: the role of stimulus structure in recognition and classification. *Mem. Cogn.* 26 146–160. 10.3758/BF03211378 9519705

[B21] Peer P. (1999). *CVL Face Database.* Available at: http://www.lrv.fri.uni-lj.si/facedb.html

[B22] Richler J. J. Cheung O. S. Gauthier I. (2011a). Beliefs alter holistic face processing…if response bias is not taken into account. *J. Vis.* 11 1–13. 10.1167/11.13.17 PMC3354002 22101018

[B23] Richler J. J. Cheung O. S. Gauthier I. (2011b). Holistic processing predicts face recognition. *Psychol. Sci.* 22 464–471. 10.1177/0956797611401753 21393576 PMC3077885

[B24] Richler J. J. Floyd R. J. Gauthier I. (2014). The vanderbilt holistic face processing test: a short and reliable measure of holistic face processing. *J. Vis.* 14:10 10.1167/14.11.10 PMC4528459 25228629

[B25] Richler J. J. Floyd R. J. Gauthier I. (2015). About-face on face recognition ability and holistic processing. *J. Vis.* 15 1–12. 10.1167/15.9.15 PMC4581616 26223027

[B26] Richler J. J. Gauthier I. (2013). When intuition fails to align with data: a reply to Rossion (2013). *Vis. Cogn.* 21 254–276. 10.1080/13506285.2013.796035 PMC3845673 24307858

[B27] Richler J. J. Gauthier I. (2014). A meta-analysis and review of holistic processing. *Psychol. Bull.* 140 1281–1302. 10.1037/a0037004 24956123 PMC4152424

[B28] Richler J. J. Mack M. L. Palmeri T. J. Gauthier I. (2011c). Inverted faces are (eventually) processed holistically. *Vis. Res.* 51 333–342. 10.1016/j.visres.2010.11.014 21130798

[B29] Richler J. J. Palmeri T. J. Gauthier I. (2012). Meanings, mechanisms, and measures of holistic processing. *Front. Psychol.* 3:553 10.3389/fpsyg.2012.00553 PMC3520179 23248611

[B30] Ross D. A. Richler J. J. Gauthier I. (2015). Reliability of composite task measurements of holistic face processing. *Behav. Res. Methods* 47 736–743. 10.3758/s13428-014-0497-4 24961957 PMC4276735

[B31] Rossion B. (2013). The composite face illusion: a whole window into our understanding of holistic face perception. *Vis. Cogn.* 21 139–253. 10.1080/13506285.2013.772929

[B32] Rossion B. Boremanse A. (2008). Nonlinear relationship between holistic processing of individual faces and picture-plane rotation: evidence from the face composite illusion. *J. Vis.* 8:3 10.1167/8.4.3 18484842

[B33] Royer J. Blais C. Gosselin F. Duncan J. Fiset D. (2015). When less is more: impact of face processing ability on recognition of visually degraded faces. *J. Exp. Psychol. Hum. Percept. Perform.* 41 1179–1183. 26168140 10.1037/xhp0000095

[B34] Salthouse T. A. Meinz E. J. (1995). Aging, inhibition, working memory, and speed. *J. Gerontol. Ser. B Psychol. Sci. Soc. Sci.* 50 297–306. 10.1093/geronb/50B.6.P297 7583809

[B35] Shilling V. M. Chetwynd A. Rabbitt P. M. (2002). Individual inconsistency across measures of inhibition: an investigation of the construct validity of inhibition in older adults. *Neuropsychologia* 40 605–619. 10.1016/S0028-3932(01)00157-9 11792402

[B36] Solina F. Peer P. Bategelj B. Juvan S. Kovač J. (2003). Color-based face detection in the “15 seconds of fame” art installation. *Paper Presented at the Conference on Computer Vision/Computer Graphics Collaboration for Model-Based Imaging Rendering, Image Analysis and Graphical Special Effects*, Roquencourt.

[B37] Tanaka J. W. Kaiser M. D. Butler S. Le Grand R. (2012). Mixed emotions: holistic and analytic perception of facial expressions. *Cogn. Emot.* 26 961–977. 10.1080/02699931.2011.630933 22273429

[B38] Thompson B. (1994). Guidelines for authors. *Educ. Psychol. Meas.* 54 834–847.

[B39] Todorov A. Loehr V. Oosterhof N. N. (2010). The obligatory nature of holistic processing of faces in social judgments. *Perception* 39 514–532. 10.1068/p6501 20514999

[B40] Underwood B. J. (1957). Interference and forgetting. *Psychol. Rev.* 64 49–60. 10.1037/h0044616 13408394

[B41] VanGulick A. E. McGugin R. W. Gauthier I. (2016). Measuring nonvisual knowledge about object categories: the semantic vanderbilt expertise test. *Behav. Res. Methods* 48 1178–1196. 10.3758/s13428-015-0637-5 26276518 PMC4754162

[B42] Ward G. Roberts M. Phillips L. (2001). Task-switching costs, Stroop-costs, and executive control: a correlational study. *Q. J. Exp. Psychol.* 54 491–511. 10.1080/713755967 11394058

[B43] Wetcher-Hendricks D. (2006). Adjustments to the correction for attenuation. *Psychol. Methods* 11 207–215. 10.1037/1082-989X.11.2.207 16784339

[B44] Wiese H. Komes J. Schweinberger S. R. (2013). Ageing faces in ageing minds: a review on the own-age bias in face recognition. *Vis. Cogn.* 21 1337–1363. 10.1080/13506285.2013.823139

[B45] Wright D. B. Stroud J. N. (2002). Age differences in lineup identification accuracy: people are better with their own age. *Law Hum. Behav.* 26 641–654. 10.1023/A:1020981501383 12508699

[B46] Yehene E. Meiran M. (2007). Is there a general task switching ability? *Acta Psychol.* 126 169–195. 10.1016/j.actpsy.2006.11.007 17223059

[B47] Young A. W. Hellawell D. Hay D. C. (1987). Configurational information in face perception. *Perception* 16 747–759. 10.1068/p160747 3454432

[B48] Yovel G. Wilmer J. B. Duchaine B. (2014). What can individual differences reveal about face processing? *Front. Hum. Neurosci.* 8:562 10.3389/fnhum.2014.00562 PMC4137541 25191241

[B49] Zebrowitz L. A. Franklin R. G. Jr. Palumbo R. (2015). Ailing voters advance attractive congressional candidates. *Evol. Psychol.* 13 16–28. 10.1177/147470491501300102 25562113 PMC4353482

[B50] Zhao M. Hayward W. G. (2010). Holistic processing underlies gender judgments of faces. *Attent. Percept. Psychophys.* 72 591–596. 10.3758/APP.72.3.591 20348564

